# Benchmark Glycan Profile of Therapeutic Monoclonal Antibodies Produced by Mammalian Cell Expression Systems

**DOI:** 10.1007/s11095-023-03628-4

**Published:** 2023-11-01

**Authors:** Shen Luo, Baolin Zhang

**Affiliations:** Office of Biotechnology Products, Office of Pharmaceutical Quality, Center for Drug Evaluation and Research, Food and Drug Administration, Silver Spring, MD 20993 USA

**Keywords:** benchmark glycan profile, biologics, glycosylation, monoclonal antibodies (mabs), quality control

## Abstract

**Purpose:**

This study aims to establish a benchmark glycan profile for commercial therapeutic monoclonal antibodies (mAbs) approved by the US Food and Drug Administration (FDA).

**Methods:**

We conducted a rigorous comparison of glycosylation data from the regulatory submissions for FDA-approved therapeutic antibodies up to May 2023. This analysis includes over 150 mAbs produced by various mammalian cell expression systems.

**Results:**

The study identified nine prevalent glycan epitopes across all FDA-approved monoclonal antibodies produced by different expression systems. These epitopes include terminal N-acetylglucosamine, core fucose, terminal galactose, high mannose, α-galactose, terminal α2,3-linked N-acetylneuraminic acid, terminal α2,6-linked N-glycolylneuraminic acid, triantennary structure, and bisecting N-acetylglucosamine, thus establishing a benchmark glycan profile.

**Conclusions:**

The findings of this study have significant implications for therapeutic antibody development, quality control, and regulatory compliance. The benchmark glycan profile enables the assessment of glycosylation consistency and comparability across a diverse range of antibody products, ensuring improved product quality within the biopharmaceutical industry.

**Supplementary Information:**

The online version contains supplementary material available at 10.1007/s11095-023-03628-4.

## Introduction

Glycosylation is a post-translational modification process that involves the addition of carbohydrate structures to proteins, including therapeutic monoclonal antibodies (mAbs) [1, 2]. The carbohydrate structures added during glycosylation can significantly influence the properties of these antibodies, including their Fc effector functions, pharmacokinetics, and immunogenicity [2, 3]. Therefore, understanding and controlling the glycosylation patterns is essential to maintain product quality and consistency during manufacturing [4].

Glycosylation is a naturally occurring process with considerable heterogeneity due to its non-template driven biosynthesis machinery [5, 6]. It can be influenced by multiple factors, such as cellular expression systems, culture conditions, and purification schemes [4]. For example, the choice of cell line, such as Chinese hamster ovary (CHO) cells or murine myeloma cells NS0 and Sp2/0, can impact the glycosylation patterns of the produced mAbs [7, 8]. Additionally, factors like media composition, growth conditions, and process parameters can also influence glycosylation [4, 9]. Consequently, glycosylation patterns or relative abundance of each glycan species, may vary between batches, leading to variations in product quality. Therefore, glycosylation is recognized as a critical quality attribute (CQA) for specific therapeutic antibodies, including both novel mAbs and biosimilars [10].

In this study, we aimed to comprehensively analyze the glycosylation patterns of United States Food and Drug Administration (FDA)-approved mAbs through an in-depth examination of release specifications and characterization data. By conducting this extensive analysis, we sought to establish a benchmark glycan profile that represents prevalent glycan structures and their respective abundances within the mAb dataset. Our analysis identified several glycan epitopes that are commonly observed in FDA-approved mAbs, including core fucose, terminal N-acetylglucosamine, terminal galactose, high mannose, terminal α2,3-linked N-acetylneuraminic acid, terminal α2,6-linked N-glycolylneuraminic acid, bisecting N-acetylglucosamine, α-galactose, and triantennary structure. These glycan epitopes have been reported to influence various aspects of mAb function, such as antibody-dependent cellular cytotoxicity (ADCC), complement-dependent cytotoxicity (CDC), and serum half-life [11–13]. The establishment of the benchmark glycan profile serves as a crucial reference point for mAb development, quality control, and regulatory compliance.

## Methods

A total of 157 therapeutic recombinant antibody products that received FDA approval up to May 2023 were compiled using Microsoft Excel (supplement Table 1). This compilation was based on an internal product list that tracks Biologic License Applications (BLAs) for biotechnology products, maintained at the FDA’s Office of Biotechnology Products (OBP). The necessary information, including the antibody type, expression system, and IgG subclass, was extracted from public databases including the FDA Label database (http://fdalabel.fda.gov/fdalabel-r/ui/search) and the Antibody Therapeutics Product Data database managed by the Antibody Society (https://www.antibodysociety.org/antibody-therapeutics-product-data/).

More detailed information, including the release specification for glycosylation and N-glycan profiles, were obtained from the drug substance release specifications and characterization section of the electronic common technical document (eCTD) for the BLAs. The majority of the glycan profiles were derived from characterization data obtained from individual reference standard lots, with only a few being averages from multiple drug substance batches.

For N-glycan profiling, the products were initially grouped based on the three expression systems (CHO, NS0, Sp2/0). Within the CHO-produced products group, further sub-grouped profiles were created for typical IgG1 mAbs, other types of IgG1s, non-IgG1 antibodies, and IgG1 Fc-fusion proteins. The percentage values of relative abundance for individual glycan species were rounded to whole numbers above 1%, to one decimal place for values between 0.5% and 1%, and to zero for values less than 0.5%. With each antibody group, the individual glycan species were sorted based on their mean relative abundance. The top ten most abundant glycans were then visually represented in a scatter plot using GraphPad Prism 9.5.1. The N-glycan structures were drawn following the Symbol Nomenclature for Glycans (SNFG) guidelines [14]. It’s worth noting that this study did not cover O-glycosylation, as it is rarely present in therapeutic antibodies except for Fc-fusion proteins.

## Results

### The Evolving Landscape of FDA-Approved Therapeutic Antibodies

**Fig. 1:**
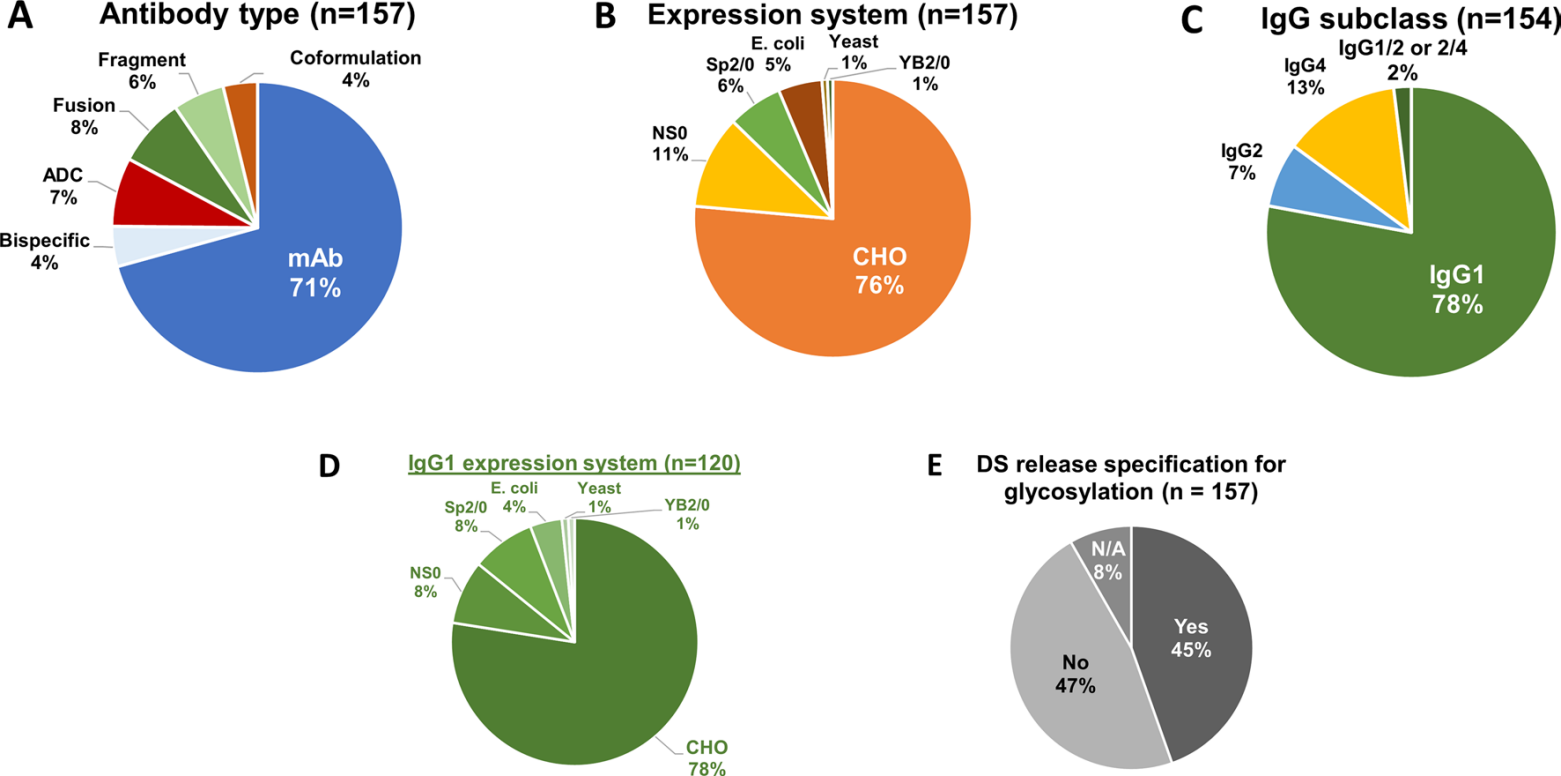
Overview of FDA-approved therapeutic monoclonal antibodies (mAbs). Analysis of 157 FDA-approved therapeutic mAbs (as of May 2023) based on (**A**) antibody type, (**B**) expression system, (**C**) IgG subclass, (**D**) IgG1 expression system, and (**E**) drug substance release specification for glycosylation. *Note: Three Fab-fragment products were excluded from the analysis in Panel C.

Over the past two decades, the landscape of therapeutic antibodies has experienced dynamic transformations and significant progress. As of May 2023, the FDA has approved a total of 157 therapeutic antibodies to treat various diseases, including cancer, autoimmune disorders, and infectious diseases. Most of these antibodies are IgG monoclonal antibodies (mAbs), making up 71% (n = 111) of the approvals. Other categories include bispecific antibodies (4%), antibody–drug conjugates (ADCs) (7%), Fc-fusion proteins (8%), IgG Fab or Fc fragments (6%), and coformulation products (4%) which consist of fixed-dose combinations of mAbs and enzymes or other mAbs (Fig. 1A).

One significant development in the field of therapeutic antibodies is the enactment of the Biologics Price Competition and Innovation Act of 2009 (BPCI Act), which has led to the approval of 27 antibody biosimilars under Sect. 351(k) of the Public Health Service Act (PHS Act). These biosimilars have demonstrated high similarities to their reference products and have shown no clinically meaningful differences in terms of safety and effectiveness. In this context, a total of seven reference products have served as the basis for these biosimilar approvals. With the support of the Biosimilar User Fee Act (BsUFA), the growth of biosimilars in the biologic drugs market is expected to continue. The BsUFA provides the FDA with additional resources to facilitate a timely and efficient review process for biosimilar applications. This, in turn, encourages further investment and development of biosimilars, enhancing competition and expanding treatment options for patients.

Glycosylation of IgG antibody is critical consideration in the production of therapeutic proteins, which are manufactured using recombinant DNA technology and various cell expression systems. Among the 157 antibody therapeutics, three mammalian cell lines are predominantly used (Fig. 1B): CHO (76%), NS0 (11%), and Sp2/0 (6%). Additionally, *E. coli* cells (5%) are exclusively used for producing non-glycosylated IgG-Fab fragments, while Yeast (1%) and the rat myeloma cell line YB2/0 (1%) are also applied, but only for two products. When classifying these antibodies by IgG subclass (Fig. 1C), IgG1 constitutes the majority (78%), followed by IgG2 (7%), IgG4 (13%), and hybrid IgG1/2 and IgG2/4 (2%). However, the other three Fab fragments are not classified in the eCTD submission system. Among the largest IgG1 subclass (n = 120), most antibodies are produced using CHO (78%), NS0 (8%), and Sp2/0 (8%) cells (Fig. 1D). Clearly, CHO cells have become the standard platform to produce all subclasses and types of therapeutic antibodies.

### Consensus Glycan Profiles Across Therapeutic Antibodies

IgG antibodies bear two N-glycans at a highly conserved site, Asn-297, located in the Fc region [2]. This glycosylation, however, exhibits significant heterogeneity among different expression systems [7]. To gain insight into glycan attributes in these IgG antibody therapeutics, we analyzed the relevant information from eCTDs. First, we examined the drug substance specification section in the 157 BLAs and found that, except for 8% of non-glycosylated antibodies, nearly half of the products (45%) had glycosylation specifications for release testing of the drug substance (Fig. 1E). This is largely associated with reporting effector function (e.g., ADCC, CDC) as part of the mechanism of action (MOA) on the product labels. This correlation aligns with the crucial role of Fc glycan in IgG binding to Fcγ receptors [15, 16] and C1q [17].

**Fig. 2:**
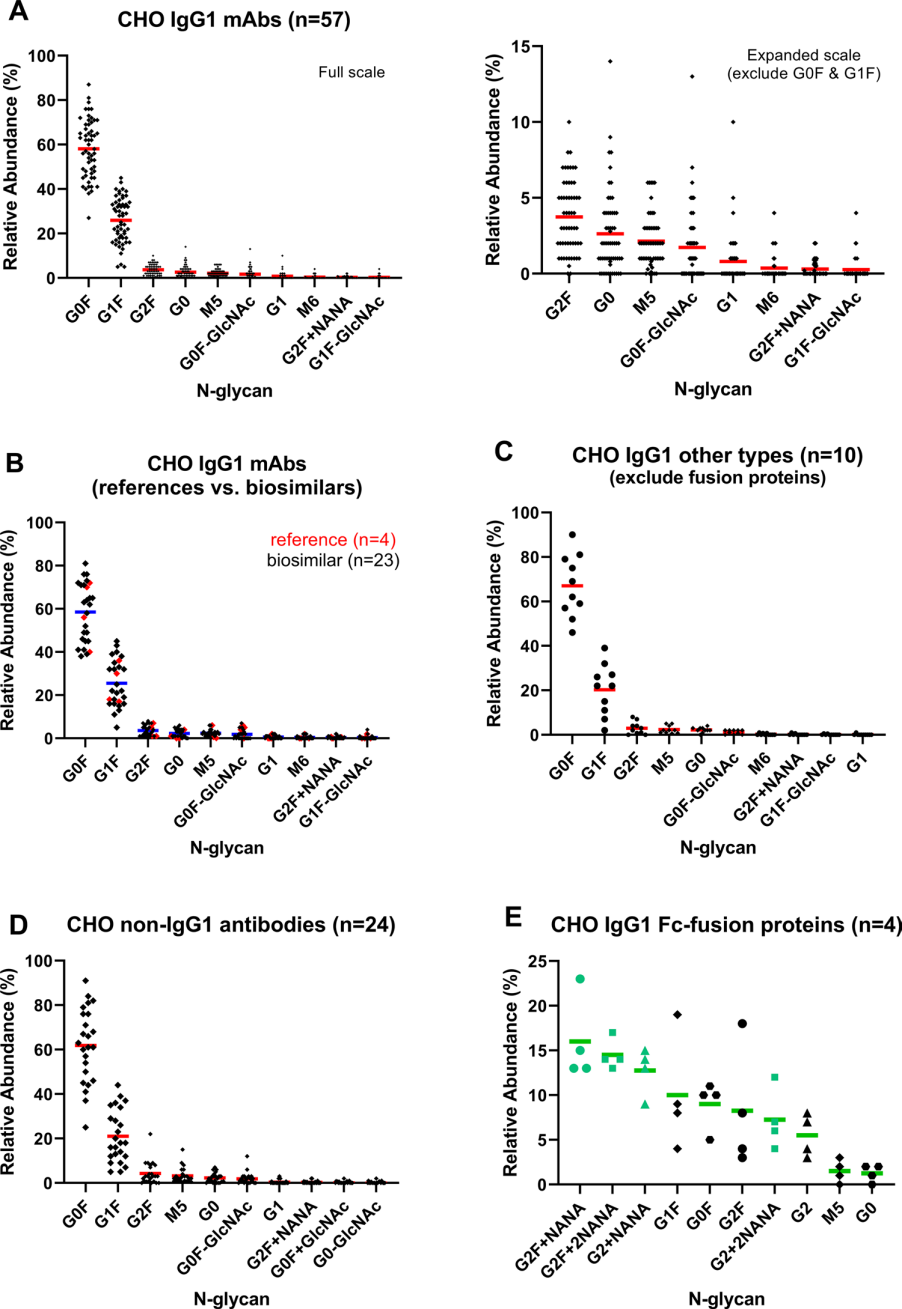
Comparative analysis of N-glycan profiles of CHO-produced mAbs. This figure presents the findings of a survey conducted on Biologic License Application (BLA) documents, focusing on N-glycan profiles of therapeutic mAbs produced in CHO cells. The majority of CHO-produced mAbs in categories A to D exhibit similar N-glycan profiles, mainly comprising G0F and G1F species. However, a notable exception is observed for IgG1-Fc fusion proteins in category E, which display a more even distribution of N-glycans, with the top three glycans being sialylated with N-Acetylneuraminic acid (NANA; green symbols). The scatter plots illustrate the mean values and distribution of the top ten N-glycans consistently found in CHO-produced mAbs in different categories: (**A**) IgG1 mAbs, (**B**) reference products (red symbols) *vs.* biosimilars of IgG1 mAbs, (**C**) other types of IgG1 mAbs including bispecific antibody (BsAb), antibody–drug conjugate (ADC), and IgG1-Fc fragment, (**D**) non-IgG1 mAbs including IgG2, IgG4, IgG1/2, and IgG2/4 mAbs, IgG4 BsAb, and IgG4 Fc-fusion protein, and (**E**) IgG1 Fc-fusion proteins. Of note, the analyses excluded antibodies in fixed-dose combination products containing more than one active pharmaceutical ingredient (API), and antibodies with unique glycan profiles designed intentionally, such as low levels of fucosylation, to ensure robust and accurate comparisons.

Next, we extracted quantitative N-glycan profiles from 119 eCTDs for antibodies produced by the top three cell lines: CHO (n = 95), NS0 (n = 16), and Sp2/0 (n = 8). The focus of this analysis was on common glycan profiles, and we excluded certain products, including: (i) antibodies produced by the other three cell lines (n = 10); (ii) antibodies in coformulation products containing more than one active pharmaceutical ingredient (API), including enzyme API (n = 6); and (iii) antibodies with unique N-glycan profiles intentionally engineered, such as non-glycosylated (n = 5), afucosylated (n = 4), and low fucosylated (n = 3) antibodies [18]. For the remaining 119 samples, we grouped them by expression system. The largest group was CHO (n = 95), which was further divided into five subgroups, as shown in Fig. 2: (A) typical IgG1 mAbs (n = 57), (B) IgG1 reference products (n = 4) and biosimilars (n = 23) of the typical mAb type, (C) other types of IgG1s, including ADCs, bispecific antibodies, and Fc fragments (n = 10), (D) non-IgG1 antibodies encompassing all types of IgG2, IgG4, IgG1/2, and IgG2/4 products (n = 24), and (E) IgG1 Fc-fusion proteins (n = 4).

**Fig. 3:**
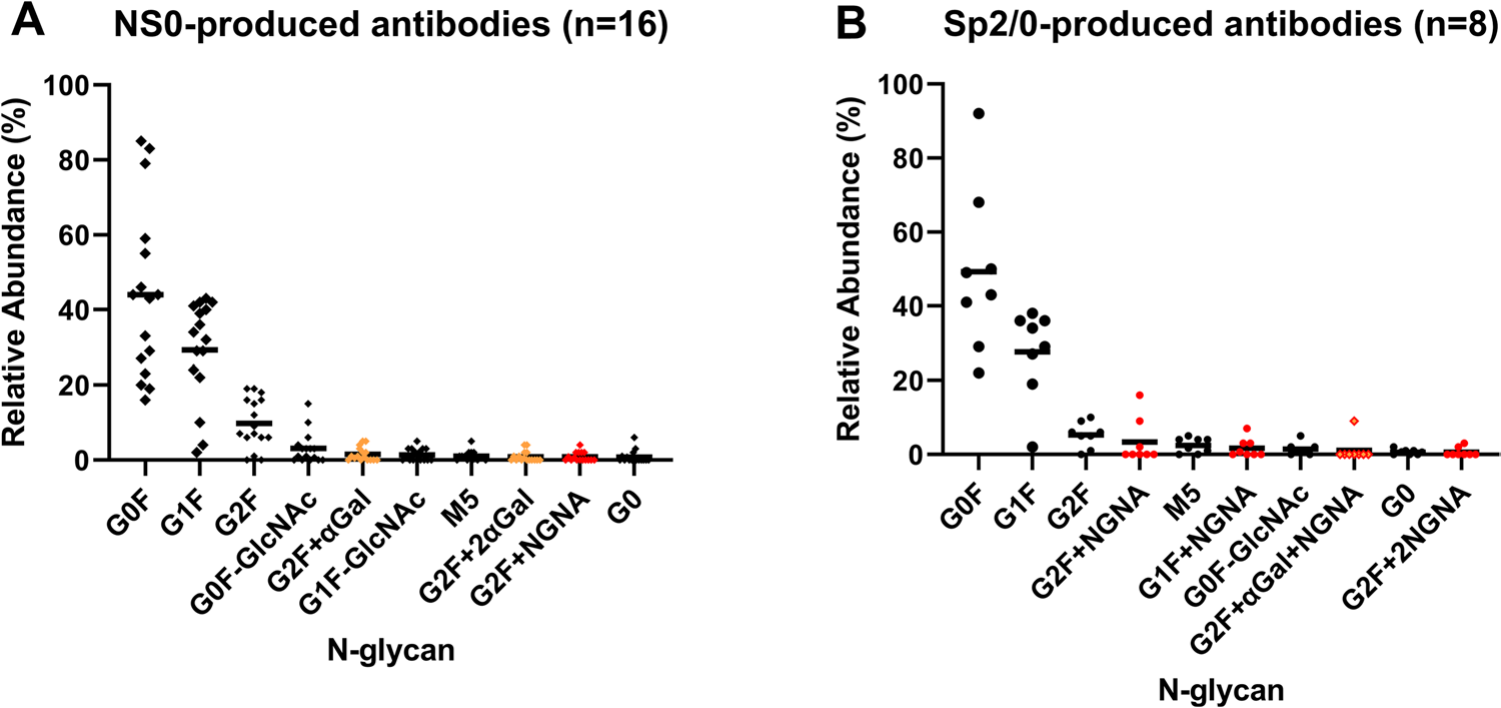
N-glycan profiles of mAbs produced by mouse myeloma cell lines. The BLA survey revealed that the most abundant N-glycans of mAbs and ADCs produced by mouse myeloma cell lines (NS0 and Sp2/0) are G0F, G1F, and G2F (A, B), like CHO-produced mAbs. However, mAbs produced in NS0 and Sp2/0 cells contain potentially immunogenic glycans, namely glycans with terminal α-Galactose (α-Gal; orange symbols) and N-Glycolylneuraminic acid (NGNA; red symbols). The scatter plots display the mean values and distribution of the top ten N-glycans that are consistently present in FDA-approved mAbs and ADCs produced in NS0 (**A**) and Sp2/0 cells (**B**).

**Fig. 4:**
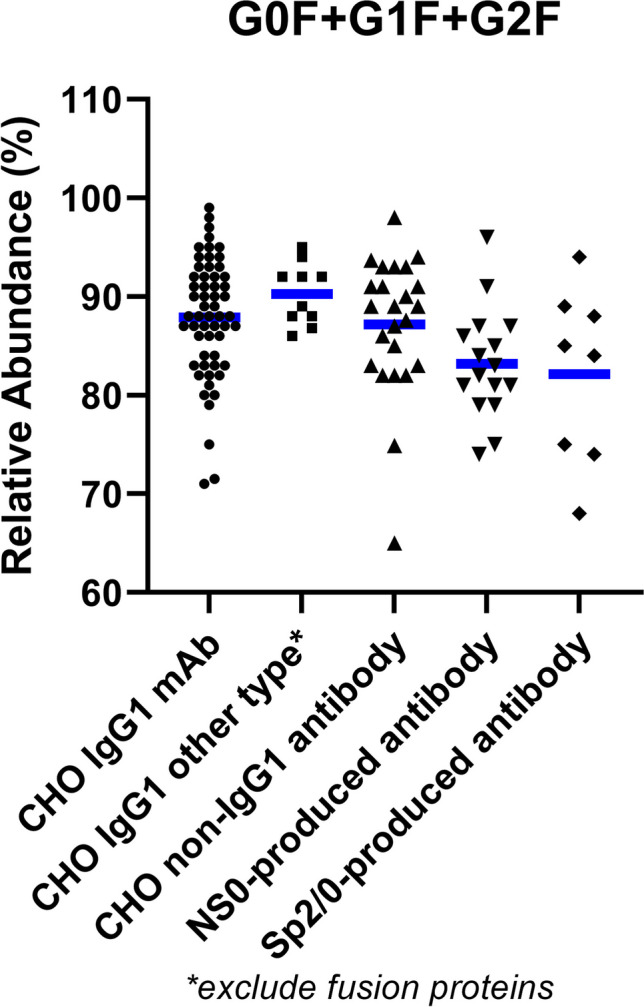
Relative abundances of the top three N-glycans in therapeutic mAbs. The scatter plots illustrate the mean values for the total abundance of the top three N-glycans (G0F, G1F, and G2F) in antibodies produced by CHO, NS0, and Sp2/0 cells. The mean values fall within a narrow range of 82–90%, while the distributions are wide, ranging 65–99%. Fusion proteins were excluded from this analysis due to their intentionally engineered glycosylation patterns.

Based on the analysis of the mean value of each N-glycan species in different groups, we have identified the top ten most abundant glycans present across antibodies produced by the top three cell lines (Figs. 2 and 3). Regardless of the expression system, these abundant glycans are predominantly biantennary complex type (Table 1). Among them, the most common N-glycans across all groups are fucosylated G0F, G1F, and G2F, being the top three N-glycans for all groups (Table 1 and Figs. 2A-D and 3), except for the Fc-fusion proteins (Fig. 2E). Similar patterns are also observed between the CHO-produced IgG1 mAb reference products and their biosimilars (Fig. 2B). While the abundance of each glycan species varies widely among products (Fig. 2A), their combined mean values when counting the top three glycans together (G0F + G1F + G2F) are within a narrow range of 82–90% across all groups (Fig. 4). Interestingly, a wide range of G0F levels has been reported to one legacy product during its long manufacturing history involving multiple process changes. Therefore, there is a possibility that such wide distributions of the top three glycans among different products are more influenced by different bioprocesses (e.g., media and culture conditions) rather than the products or expression systems themselves. One example is the observation of rapid shift from G0F to G1F on a CHO-produced mAb when increasing concentrations of galactosylation substrates (uridine, manganese chloride, and galactose) in the feed medium [19].

**Table 1:**
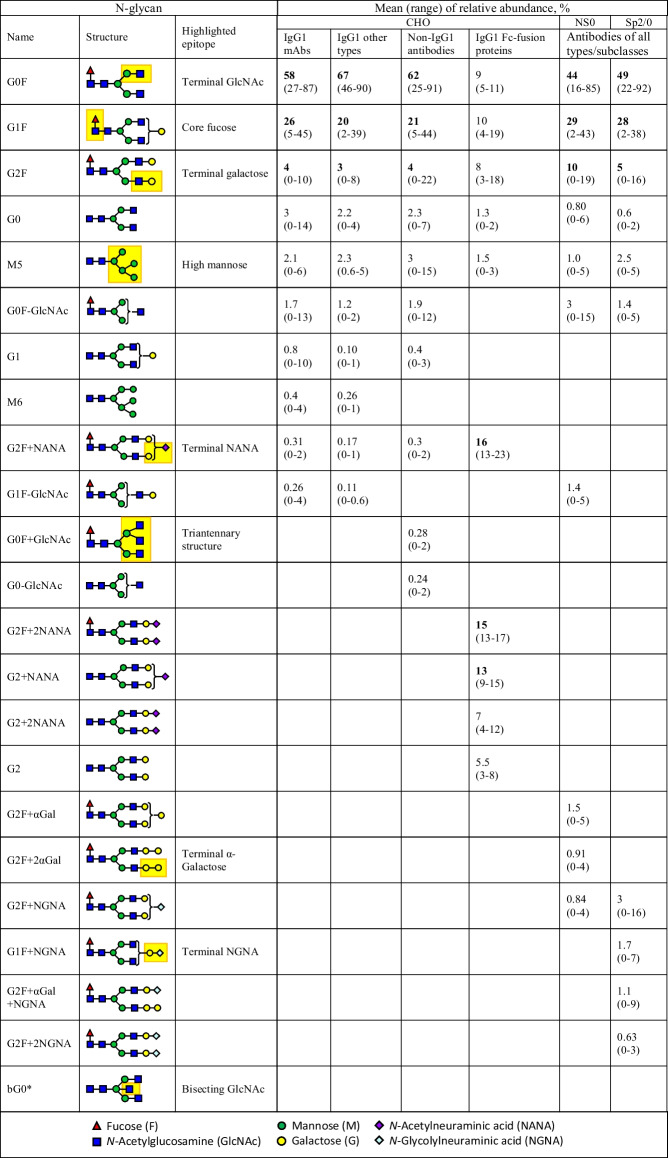
Characterization of top ten N-Glycan structures and terminal epitopes in FDA-approved monoclonal antibodies: cell line-specific distribution and relative abundances. This table provides a summary of the glycan structures and their corresponding terminal epitopes, along with the mean percentage relative abundances and distribution ranges, for the top ten N-glycans found in FDA-approved monoclonal antibodies. The antibodies were produced in different cell lines, including CHO (57 IgG1 mAbs, 10 other type IgG1s, 24 non-IgG1s, and 4 IgG1 Fc-fusion proteins), NS0 (n = 16), and Sp2/0 (n = 8). The glycan data is sorted based on IgG1 mAbs, and the top three mean values within each group are highlighted in **bold** fonts. The analysis excludes antibodies in fixed-dose combination products and those with unique glycan profiles due to specific design characteristics, such as low levels of fucosylation. Percentage values less than 0.5% were rounded to zero

* Reported in several products at low levels, but at high levels in one glycol-engineered product.

In addition to the top three glycans, there are other glycans present at lower abundance with mean values not exceeding 3%, such as afucosylated G0 and M5, which are among the top ten glycans in all groups, including Fc-fusion proteins (Table 1, Figs. 2 and 3). Furthermore, fucosylated G0F-GlcNAc is also on the top-ten lists for all antibodies except for the Fc-fusion proteins (Table 1, Figs. 2A-D and 3). High mannose glycans, represented by M5 and M6, have an average abundance of 1–3% and below 0.5%, respectively, across all antibodies (Table 1). This indicates that the levels of high mannose glycans are generally low, likely due to optimized bioprocesses, which help minimize their impact on the efficacy and pharmacokinetics of the product [20].

### Distinct Glycan Profiles in Some Therapeutic Antibodies

Despite six common glycans, there are two main differences in N-glycan profiles among these antibodies. Firstly, as mentioned above, Fc-fusion proteins exhibit distinct top-three glycans compared to other types of antibodies. This variation arises from the presence of multiple N-glycosylation sites in the non-Fc region of the protein, leading to attachments of different glycans compared to the Fc-glycans. For example, it has been reported that the extracellular domain of the tumor necrosis factor receptor on etanercept (Enbrel®) carries abundant sialylated N-glycans, which are not commonly seen in neutral Fc-glycans [21, 22]. Consequently, the top three glycans in Fc-fusion proteins are all sialylated: G2F + NANA, G2F + 2NANA, and G2 + NANA. Additionally, the top ten glycans are more evenly distributed compared to glycans in other types of antibodies (Fig. 2E).

Secondly, differences are also observed on glycans with non-human epitopes, which could potentially trigger an immune response. Specifically, two potentially immunogenic epitopes, namely α-galactose (Galα(1,3)Galβ(1,4)GlcNAc) and sialic acid N-glycolylneuraminic acid (NGNA), show notable differences. The α-galactose epitope is mainly detected in NS0-produced antibodies (G2F + αGal, G2F + 2αGal, and G2F + NGNA), while the NGNA epitope is mainly found in Sp2/0-produced antibodies (G2F + NGNA, G1F + NGNA, G2F + αGal + NGNA, and G2F + 2NGNA) (Fig. 3). Of note, Fig. 3B excludes a unique case, cetuximab (Erbitux®), an Sp2/0-produced IgG1 mAb reported to have a second N-glycosylation site in the Fab region carrying abundant α-galactose and NGNA glycans, which are different from its Fc-glycans [23]. In contrast, CHO-produced antibodies predominantly carry human N-acetylneuraminic acid (NANA) glycans (see Fig. 2), and their levels are generally very low, in average less than 0.5%, except for Fc-fusion proteins.

### A Benchmark Glycan Profile Across Therapeutic IgG mAbs

Despite the extensive structural diversity observed, the top ten glycans found in all groups share eight common terminal epitopes, which are highlighted on glycan structures in Table 1. These eight epitopes consist of non-immunogenic ones: core fucose, terminal N-acetylglucosamine (GlcNAc), terminal galactose, high mannose, triantennary structure, and terminal NANA with an exclusive α2,3-linkage on IgG1 monoclonal antibodies produced in CHO cells [24]. Additionally, there are two potentially immunogenic epitopes, namely α-galactose and terminal NGNA with mostly α2,6-linkage [25–27]. Moreover, the bisecting GlcNAc epitope (Table 1) was detected at very low level in several products but was found at a significantly high level in one glyco-engineered IgG1 mAb, mainly afucosylated [18]. It is noteworthy that afucosylated mAbs demonstrate much higher binding affinity to Fcγ receptors compared to fucosylated mAbs, resulting in enhanced ADCC effects [28].

## Discussion

In this study, we aimed to provide a comprehensive understanding of mAb glycosylation by analyzing glycosylation data from FDA-approved biologics up to May 2023. Our analysis covered more than 150 mAbs produced through diverse mammalian cell expression systems (Supplement Table 1), enabling us to establish a benchmark glycan profile for therapeutic antibodies (Figs. 2 and 3). The identification of nine prevalent glycan epitopes commonly found in mAbs represents a significant contribution to the field. These epitopes, including terminal N-acetylglucosamine, core fucose, terminal galactose, high mannose, α-galactose, terminal α2,3-linked N-acetylneuraminic acid, terminal α2,6-linked N-glycolylneuraminic acid, triantennary structure, and bisecting N-acetylglucosamine, provide valuable insights into the glycosylation landscape of therapeutic mAbs (Table 1). This information is vital for assessing and monitoring glycosylation patterns during mAb production, ensuring consistent product quality, and mitigating the risk of undesirable glycan profiles that may lead to altered pharmacokinetics or immunogenicity.

It is important to emphasize the significance of employing rigorous glycosylation analysis and quality control throughout the entire mAb manufacturing process. Standardized analytical methods are essential to obtain accurate and reliable glycosylation data, allowing for a better understanding of the impact of glycosylation on mAb functionality and safety [2, 10, 29]. The benchmark glycan profile presented in this study can serve as a valuable reference for assessing the glycosylation of biosimilars to FDA-approved mAbs. This will streamline the regulatory approval process and ensure the comparability of biosimilars with their reference products, further facilitating the development of novel therapeutics.

Our findings also highlight the influence of different cell lines on mAb glycosylation patterns [30]. Notably, CHO cells, with their ability to perform complex glycosylation similar to human cells, are preferred for producing mAbs with human-like glycosylation patterns (Fig. 1B). NS0 and Sp2/0 cells, despite the tendency of adding non-human terminal epitopes to some low-abundant glycans (Fig. 3), are also used to manufacture mAbs with the same predominant glycans as those on CHO-produced mAbs (Fig. 4). The established safety and regulatory track records of these cell lines make them the primary choices for therapeutic antibody production (Fig. 1B).

One critical consideration when interpreting our results is the dynamic nature of glycosylation. The glycosylation profiles of mAbs may vary depending on various factors, such as cell expression systems, cell culture conditions, manufacturing processes, and analytical methods [4]. Therefore, continuous monitoring and analysis of glycosylation patterns in different batches of mAbs are essential to maintaining consistent product quality and optimizing therapeutic effectiveness.

In conclusion, the comprehensive analysis of glycosylation data from FDA-approved biologics up to May 2023 has allowed us to establish a benchmark glycan profile for mAbs. The identification of prevalent glycan epitopes and the insights gained from this study provide a solid foundation for understanding the glycosylation landscape of mAbs. This knowledge will aid researchers and developers in producing mAbs with optimized glycosylation patterns, thus enhancing therapeutic efficacy and safety. Our work underscores the importance of rigorous glycosylation analysis and quality control in the mAb manufacturing process and highlights the impact of different cell lines on glycosylation patterns. We hope that this study will inspire further research in this field and pave the way for the continued progress of mAb-based therapies in the future.

### Supplementary Information

Below is the link to the electronic supplementary material.

Supplementary Table-1 (XLSX 23.7 KB)

## Data Availability

The authors confirm that the data supporting the findings of this study are available within the article and its supplementary materials. However, the raw data for individual products are proprietary information in the regulatory submissions and are not publicly available.

## Declarations

### Disclaimer

This article reflects the views of the authors and should not be construed to represent FDA’s views or policies.
